# Exploring priming responses involved in peach fruit acclimation to cold stress

**DOI:** 10.1038/s41598-017-11933-3

**Published:** 2017-09-12

**Authors:** Georgia Tanou, Ioannis S. Minas, Federico Scossa, Maya Belghazi, Aliki Xanthopoulou, Ioannis Ganopoulos, Panagiotis Madesis, Alisdair Fernie, Athanassios Molassiotis

**Affiliations:** 1 Department of Agriculture, Aristotle University of Thessaloniki, 54124 Thessaloniki, Greece; 2 Department of Horticulture and Landscape Architecture, Colorado State University, Fort Collins, CO USA; 3 Western Colorado Research Center at Orchard Mesa, Colorado State University, Grand Junction, CO USA; 4 Max-Planck-Institut für Molekulare Pflanzenphysiologie, 14476 Potsdam-Golm, Germany; 5 Consiglio per la ricerca in agricoltura e l’analisi dell’economia agraria, Centro di ricerca per la Frutticoltura (CREA-FRU), Roma, Italy; 6 Proteomics Analysis Center (CAPM), Faculty of Medicine, 13916 Marseilles, France; 7 Institute of Applied Biotechnology, CERTH, Thessaloniki, Greece

## Abstract

Cold storage of fruit may induce the physiological disorder chilling injury (CI); however, the molecular basis of CI development remains largely unexplored. Simulated conditions of CI priming and suppression provided an interesting experimental system to study cold response in fruit. Peaches (cv. June Gold) at the commercial harvest (CH) or tree-ripe (TR) stages were immediately exposed to cold treatment (40 d, 0 °C) and an additional group of CH fruits were pre-conditioned 48 h at 20 °C prior to low-temperature exposure (pre-conditioning, PC). Following cold treatment, the ripening behaviour of the three groups of fruits was analysed (3 d, 20 °C). Parallel proteomic, metabolomic and targeted transcription comparisons were employed to characterize the response of fruit to CI expression. Physiological data indicated that PC suppressed CI symptoms and induced more ethylene biosynthesis than the other treatments. Differences in the protein and metabolic profiles were identified, both among treatments and before and after cold exposure. Transcriptional expression patterns of several genes were consistent with their protein abundance models. Interestingly, metabolomic and gene expression results revealed a possible role for valine and/or isoleucine in CI tolerance. Overall, this study provides new insights into molecular changes during fruit acclimation to cold environment.

## Introduction

Cold is one of several important environmental stresses influencing plant performance and distribution^1^. Tolerance to low but non-freezing temperatures, a phenomenon known as cold acclimation, is a highly dynamic stress-response mechanism that involves complex cross-talk between signal transduction and gene expression^2^. In *Arabidopsis thaliana*, cold acclimation involves rapid, cold-induced expression of the C-repeat/dehydration-responsive element binding factor (CBF) transcriptional activators, followed by expression of CBF-targeted genes that orchestrate cold responses^3^.

Most fruit species experience low-temperature stress during commercial cold storage (0 to 5 °C). Stone fruits (*Prunus* spp.), including peach fruit [*Prunus persica* (L.) Batsch], are highly susceptible to CI^4^. The major symptoms of CI in peach fruit are caused by a lack of juice, flesh browning and reddening^5^. Chilling injury symptoms in peach develop during maintenance at room temperature following prolonged cold storage^6^. Understanding the mechanisms of how fruit respond to cold stress is of considerable economic and scientific interest^7^.

Despite significant progress in illuminating the molecular basis of plant acclimation to cold stress, fruit biology research in this area is lagging far behind. Recently, peach-specific transcriptional control of cold responses has received considerable attention. Almost 70% of the heat-responsive genes in peach also respond to cold in *Arabidopsis*, while several genes of the CBF pathway were transcriptionally affected in cold-stored peaches^8^. Studies using microarray and RNA-Seq analysis identified numerous cold-regulated genes in peach, including AGAMOUS-LIKE, MYB transcription factor and ALCATRAZ/SPATULA transcription factor^9, 10^. Proteomic evidence corroborates these studies by identifying peach proteins involved in cold responses, such as abscisic acid stress ripening protein, type II SK2 dehydrin, PR-1 protein and voltage-dependent anion channel (Yu *et al*.^11^; Almeida *et al*.^11^). However, much less is known about changes in peach metabolic fluxes under cold^12^. Metabolomic study using six different peach varieties revealed metabolic rearrangements related to CI development^13^. Most cold studies in peach fruit have been performed during expression of CI symptoms^14^, reflecting causal mechanisms linking such changes with damaged fruit cells. However, information about what happens before cold is scant^13^.

We hypothesize that a controlled delayed cooling (pre-conditioning) treatment just after harvest^15^ serves as a priming-dependent signaling to prepare fruit against cold stresses-induced CI symptoms. Here, we compared the effect of this pre-conditioning treatment with immediately-cooled, commercially harvested fruit or tree-ripened peaches on the cold response. We used a detailed physiological analysis to show that pre-conditioning can induce cold acclimation. By combining proteomic, metabolomic and transcriptomic approaches before and after chilling exposure, several cold-affected proteins, metabolites, genes and pathways were identified and their involvement in peach fruit acclimation to cold is discussed.

## Results

### Delayed exposure of peach fruit to cold conditions inhibits CI symptom development

**Figure 1:**
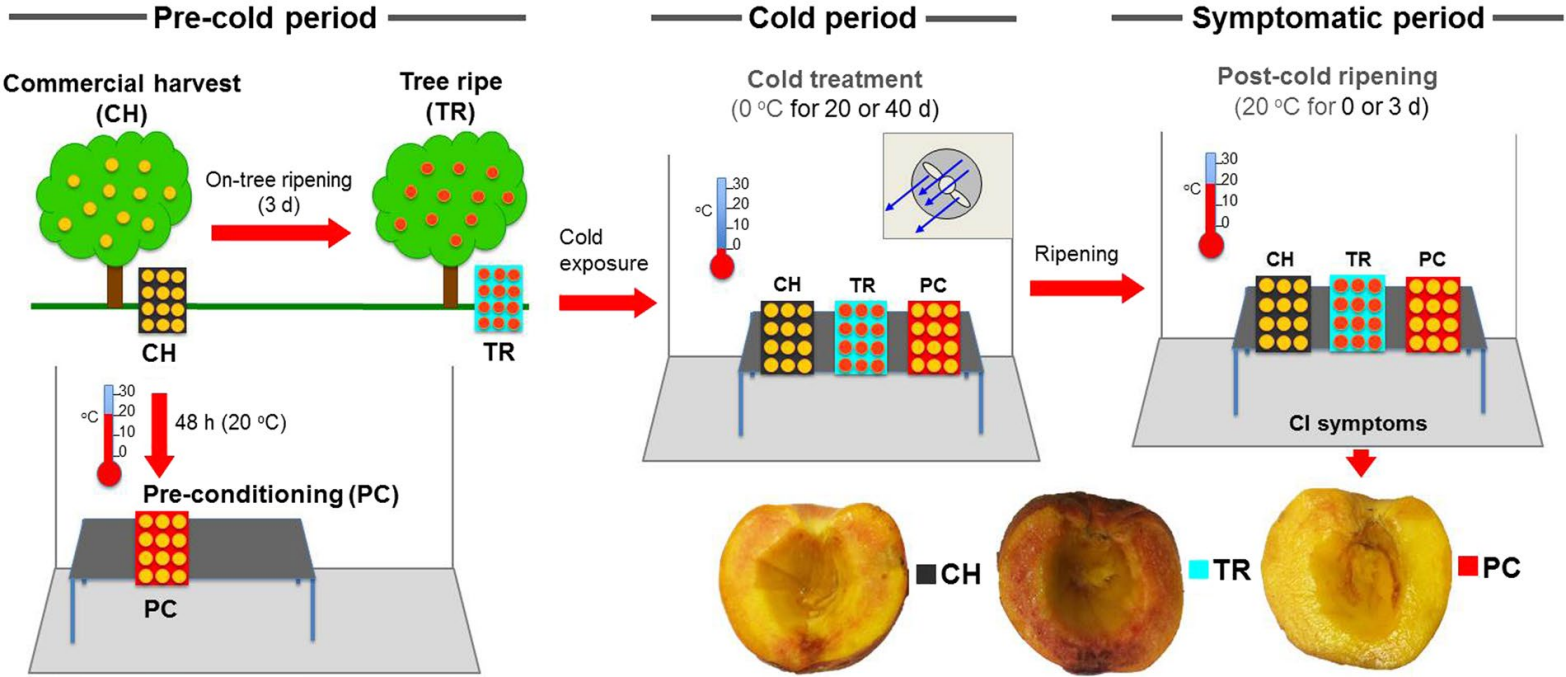
Experimental design and CI-associated peach phenotypes in response to treatments. Schematic representation of the experimental setup and conditions used for peach fruit sampling and measurements. Peaches (cv. ‘June Gold’) were harvested either at the commercial harvest (CH) or tree ripe (TR) stage and immediately transferred to cold treatment (0 **°**C) for 20 or 40 d. An additional group of CH fruits were ripened off-tree (pre-conditioning, PC) at 20 **°**C for two days prior to cold exposure. Following cold treatment, all groups of peaches (CH, TR, and PC) were ripened at 20 **°**C for up to three days. Pre-cold, cold and CI-symptomatic periods are indicated with green, blue and red lines, respectively, while arrows show transfer to the next condition. Representative peach phenotypes were observed after three days of ripening at 20 **°**C following 40 d cold treatment.

**Figure 2:**
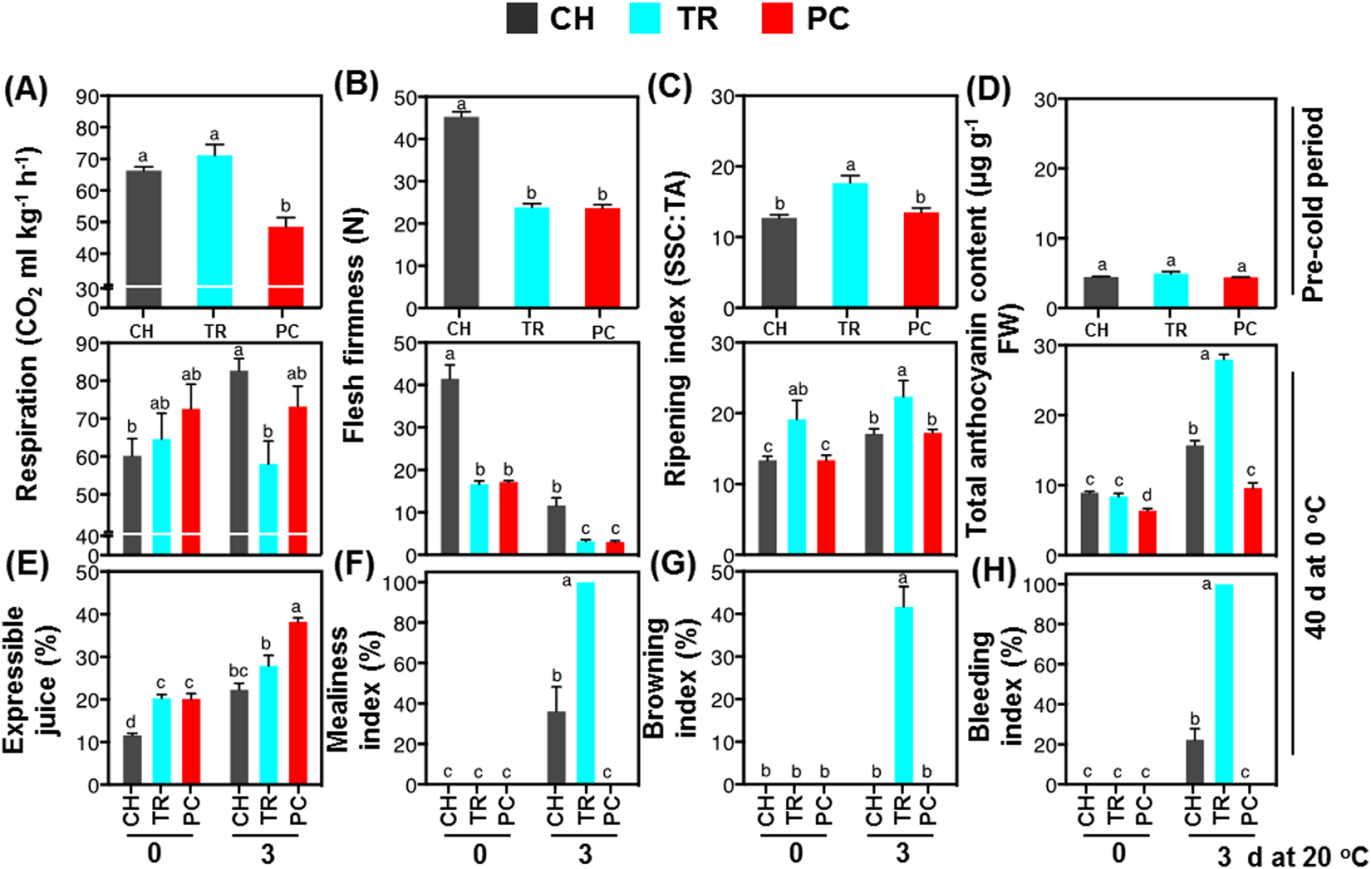
Physiological characterization of ripening and chilling injury responses of ‘June Gold’ peaches. Changes in (**A**) respiration rate, (**B**) flesh firmness, (**C**) ripening index (SSC*/*TA ratio), (**D**) total anthocyanin concentration, (**E**) expressible juice (%), (**F**) mealiness index (%), (**G**) browning index (%), and (**I**) bleeding index (%) of fruit harvested at the commercial harvest (CH) or tree ripe (TR) stages and of CH fruit pre-conditioned (PC). Physiological indicators were tested prior to cold treatment (pre-cold period), just after 40 d cold treatment at 0 °C (0 d at 20 °C), and after three days ripening at 20 °C (CI-symptomatic period). Different letters above bars indicate significant differences between treatments (*P* = 0.05) at each time. Values are means ± SE (*n* = 6).

To gain insight into the regulation of CI syndrome in peach fruit during post-cold ripening at 20 °C, we examined different harvest maturities and the effect of pre-conditioning before chilling (CH, TR, and PC) for how they affect the CI responses of peach fruit after 40 d cold exposure (Fig. 1). Fruit were harvested at two maturities based on flesh firmness (CH and TR) and immediately exposed to cold treatment (0 °C, 95% RH) conditions. An additional group of CH fruit were ripened 48 h to reach the same flesh firmness as TR fruit prior cold treatment (pre-conditioning, PC). Flesh firmness during cold treatment and subsequent ripening at 20 °C was greater in CH fruit than in TR or PC fruit, with the latter two being similar (Figs 2B and S1). PC fruit exhibited a low respiration rate at harvest prior to cold, while TR fruit had their lowest respiration after 40 d cold plus three days at 20 °C (Fig. 2A). TR fruit had a higher ripening index at harvest and during ripening at 20 °C after 20 or 40 d cold treatment (Fig. 2A and Supplementary Fig. S1). Controlled delayed cooling of peach fruit for two days after commercial harvest (PC) provided significant protection from CI development: PC fruit were CI-free after three days ripening at 20 °C following 40 d cold treatment. Fruit harvested at the commercial harvest (CH) stage and immediately stored at 0 °C expressed moderate CI symptoms after three days ripening at 20 °C following 40 d cold treatment, while tree-ripened fruits (TR) exhibited severe CI symptoms (Fig. 1). TR treatment-induced CI symptoms included high mealiness (Fig. 2F), browning (Fig. 2G) and bleeding (Fig. 2I) indices, or high concentration of anthocyanins (Fig. 2D) and reduced expressible juice (Fig. 2E). In PC fruit, trends in these measures were remarkably reversed. CI symptoms were not visible or detected by objective measurements of TR and CH fruits immediately upon removal from 40 d cold. Less-intense symptoms, but the same trend showing greater susceptibility of TR fruit to CI, were observed after 20 d cold treatment plus three days ripening at 20 °C (Supplementary Fig. S1).

### Ethylene biosynthesis during peach fruit ripening is induced by pre-conditioning

**Figure 3:**
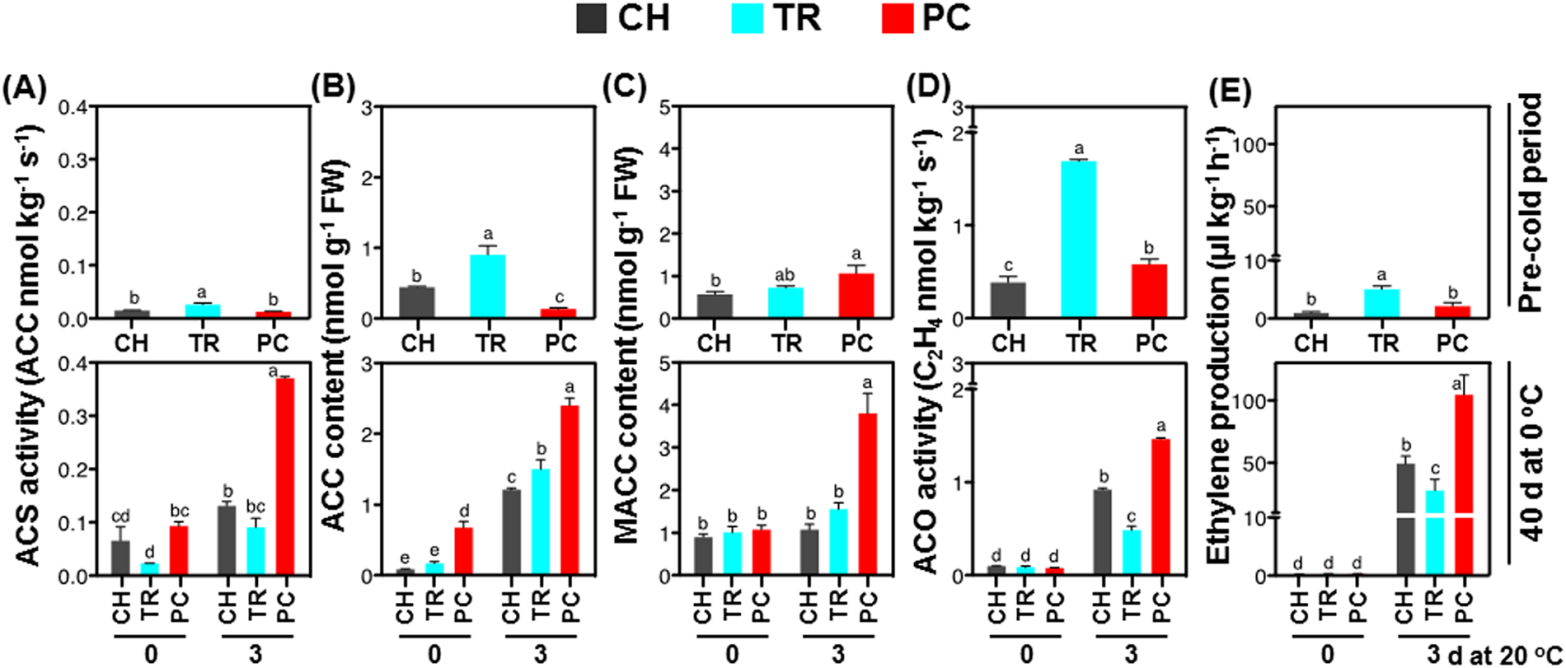
Ethylene biosynthetic pathway induction by pre-conditioning treatment in cold-stored peach fruit. Changes on ACS activity (**A**), ACC content (**B**), MACC content (**C**), ACO activity (**D**) and ethylene production (**E**) in fruit harvested at the commercial harvest (CH) or tree ripe (TR) stages and of CH fruit that was pre-conditioned (PC). Ethylene synthesis was measured prior to cold treatment (pre-cold period), just after 40 d cold at 0 °C (0 d at 0 °C), and after three days ripening at 20 °C. Additional experimental details as described (Fig. 1). Different letters above bars indicate significant differences between treatments (*P* = 0.05) at each time point. Values are means ± SE (*n* = 6).

To test whether ethylene is involved in regulating the development of CI phenotypes, GC analysis was used to measure ethylene production and selected intermediates of its biosynthesis. Before chilling, there was more ethylene synthesis (Fig. 3E), ACS and ACO enzymatic activities (Fig. 3A,D), and more ACC (Fig. 3B) in TR fruit than in other treatments; however, this was reversed during the CI symptomatic period (three days at 20 °C following 40 d at 0 °C). Although ACC accumulation was greater in PC fruit just after cold treatment (0 d at 20 °C following 40 d at 0 °C) (Fig. 3C), all treatments exhibited similarly low ethylene production at this time. Importantly, during the CI symptomatic period, PC treatment induced stronger ethylene production (Fig. 3E), greater ACS and ACO activities, and higher ACC and MACC concentrations than the other treatments (Fig. 3A–D). A similar trend was seen after three days ripening at 20 °C following 20 d cold treatment (Supplementary Fig. S2).

### Identification of proteins characterizing CI syndrome as affected by fruit pre-cold history

To characterize the molecular regulation of the different peach CI phenotypes (Fig. 1), we compared the proteome profile of mesocarp tissue after harvest but before cold treatment (CH, TR, and PC) with tissue from the CI symptomatic period (after 40 d cold plus three days ripening at 20 °C; CH 40 + 3, TR 40 + 3, and PC 40 + 3). 2DE-analysis (reference map in Supplementary Fig. S3; see also Supplementary Fig. S4) followed by mass spectrometry allowed identification of 171 proteins that changed among treatments based on the Student’s *t*-test and was further validated by the 1.5-fold change threshold (Supplementary Table S3). Identified proteins were classified into functional categories as described^16^. Detailed information regarding protein identification is presented (Supplementary Table S4).

**Figure 4:**
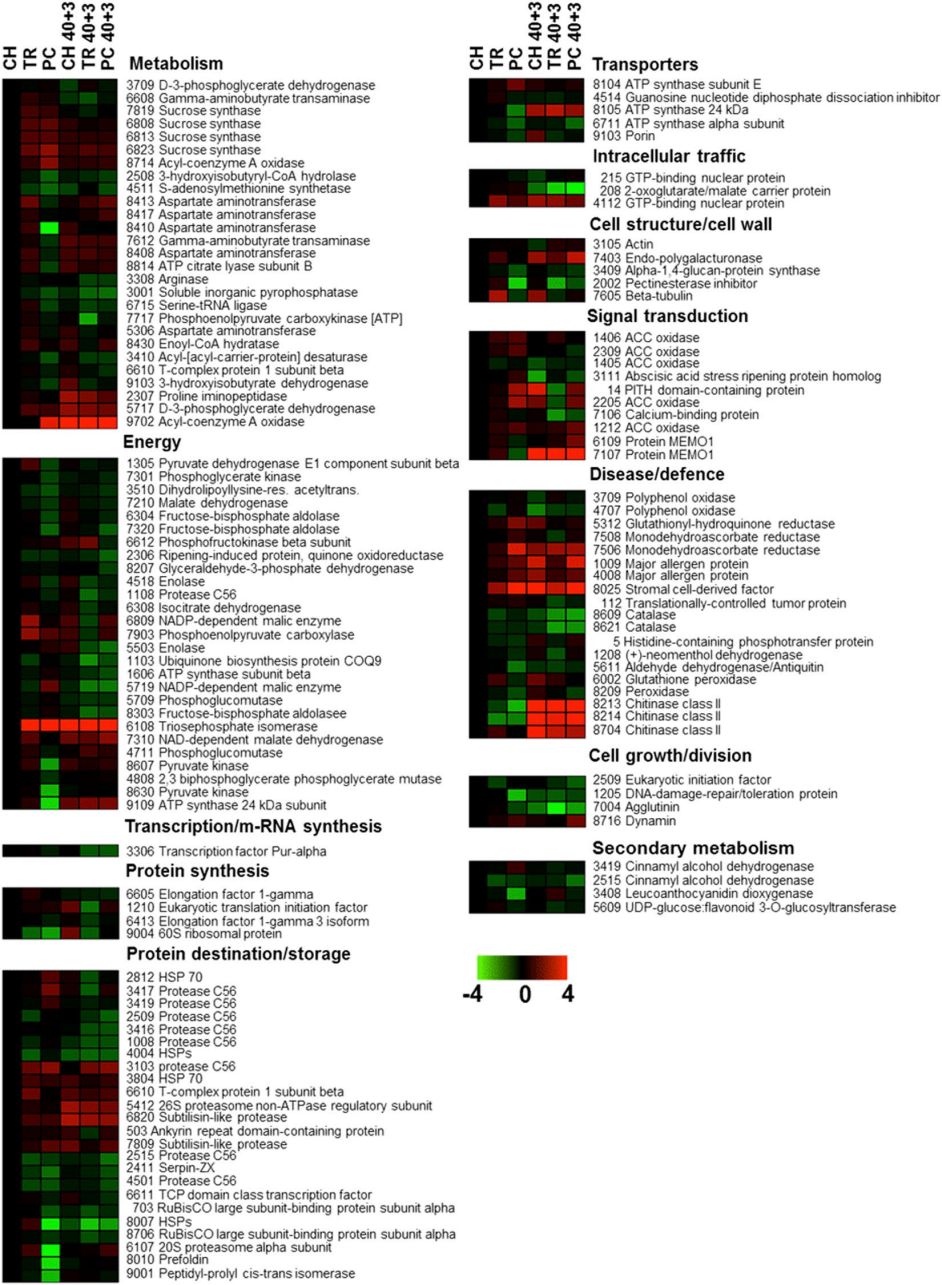
Protein abundance profiling in pre-cold and CI-symptomatic peach fruit. Heat map of proteins that demonstrated statistically significant differences among treatments. Experimental details were as described (Fig. 1). The color scale illustrates the relative abundance of each protein across the three treatments; red and green indicate enhanced and reduced abundance in TR and PC samples than in CH, respectively. The color intensity indicates the degree of protein up- or down-accumulation. The number of a protein spot corresponds to that listed (Supplementary Table S4).

**Figure 5:**
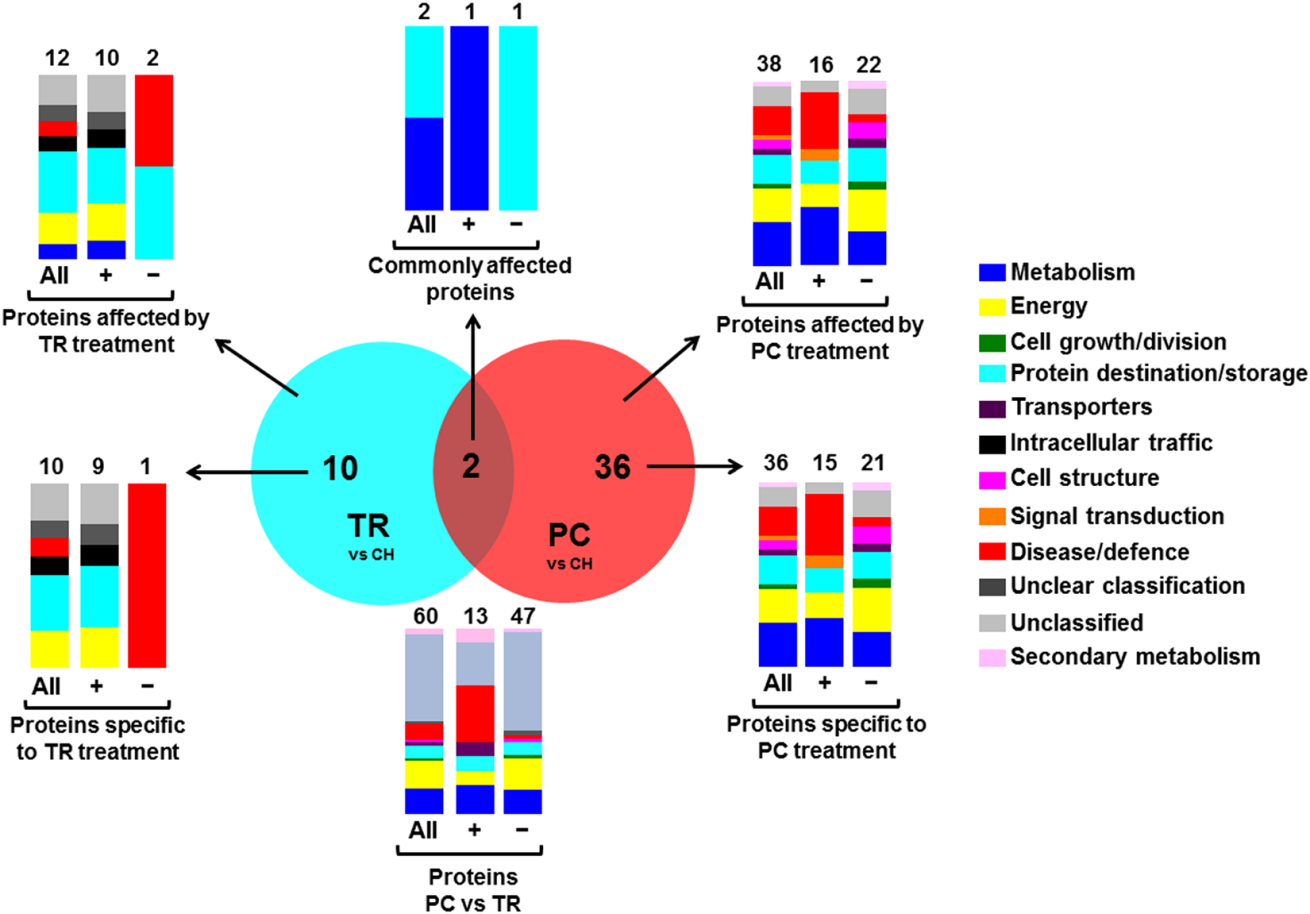
Characterization of identified peach proteins accumulated during the pre-cold period. VENN diagram showing the number of commonly and uniquely expressed proteins expressed in peach in TR and PC fruit prior to cold treatment compared to CH. Additional experimental details as described (Fig. 1). Functional classification (Bevan *et al*.^16^) of peach proteins that were commonly or specifically affected by TR and PC treatments and the numbers of total, up-accumulated (+) or down-accumulated (−) identified proteins is also indicated.

Harvest timing and postharvest handling modulated protein abundance (up regulated or down regulated) in the heat map profile (Fig. 4). Before chilling, proteomic analysis focused on three sets of proteins: i) proteins whose abundance was affected by on-tree ripening: TR treatment compared to CH (n = 12; 10 up regulated and two down regulated), ii) proteins whose abundance was affected by pre-conditioning: PC compared to CH (n = 38; 16 up regulated and 22 down regulated), and iii) proteins which differed between PC and TR fruit (n = 60; 13 up regulated and 47 down regulated; Fig. 5). Interestingly, there were only two common proteins affected by both TR and PC treatments compared to CH, while 10 proteins were affected exclusively by TR and 36 by PC. According to functional classification, the majority of proteins modulated before chilling belonged to protein destination and storage, energy, disease/defence and metabolism (Fig. 5).

**Figure 6:**
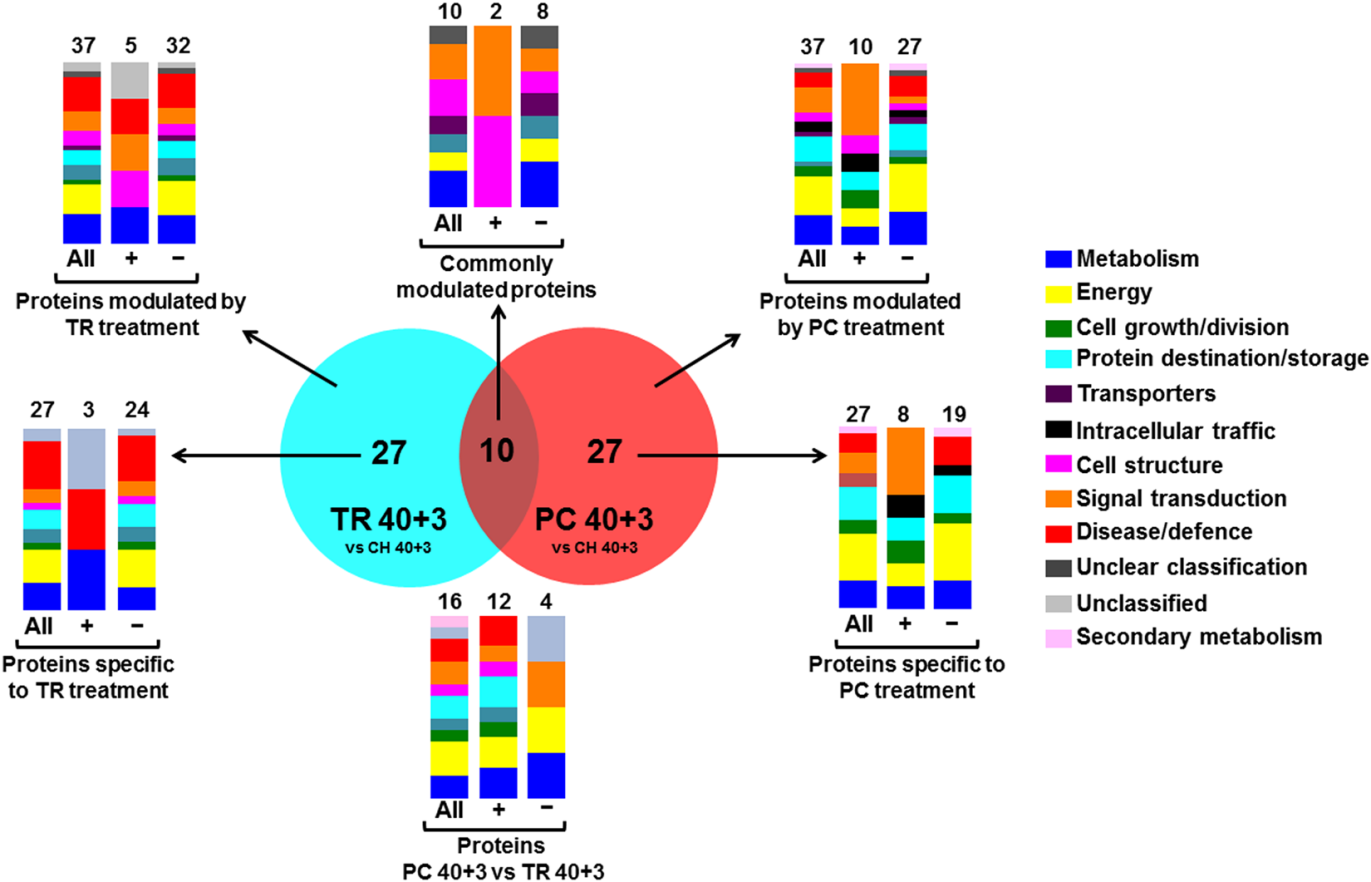
Characterization of identified peach proteins accumulated during the symptomatic period. VENN diagram showing the number of commonly and uniquely expressed proteins after three days ripening at 20 °C following 40 d cold treatment, comparing TR 40 + 3 and PC 40 + 3 fruit with CH 40 + 3. Additional experimental details were as described (Fig. 1). Functional classification of peach proteins that are commonly or specifically affected by TR and PC treatments was performed as described (Bevan *et al*.^16^; the number of altered, up-accumulated (+) or down-accumulated (−) identified proteins is also indicated.

Proteomic analysis at the CI symptomatic period (three days at 20 °C following 40 d at 0 °C) showed that: i) severely chilling-injured TR 40 + 3 fruit differed in 37 proteins (five up regulated and 32 down regulated) compared to CH 40 + 3 (moderate CI symptoms); ii) 37 proteins (10 up regulated and 27 down regulated) differed in CI-free PC 40 + 3 fruit compared to CH 40 + 3; iii) 27 proteins were exclusively modulated in TR 40 + 3 or PC 40 + 3 samples, while 10 proteins were commonly modulated by the two treatments; and iv) the differences between the two contrasting CI phenotypes, TR 40 + 3 and PC 40 + 3, affected only 16 proteins (12 up regulated and four down regulated) (Fig. 6). In all samples, the main functional categories of proteins modulated by ripening following cold treatment were energy, metabolism, protein destination and storage, signal transduction, disease/defence and cell structure (Fig. 6). Protein changes affected by cold treatment and ripening at 20 °C within treatments are also presented (Supplementary Fig. S5). Forty days cold treatment followed by three days ripening modulated 33 (26 up regulated and seven down regulated), 40 (eight up regulated and 32 down regulated) and 56 (30 up regulated and 26 down regulated) proteins in CH, TR and PC peach fruit, respectively. Fourteen, 24 and 33 proteins were modulated exclusively in CH, TR and PC fruit, respectively. More interestingly, 25 out of the 26 overlapping proteins that were commonly modulated by two (n = 3, n = 7 and n = 10) or by all three (n = 6) postharvest conditions tested responded in the same direction (up regulated or down regulated; Supplementary Fig. S5).

### Expression patterns of selected genes

**Figure 7:**
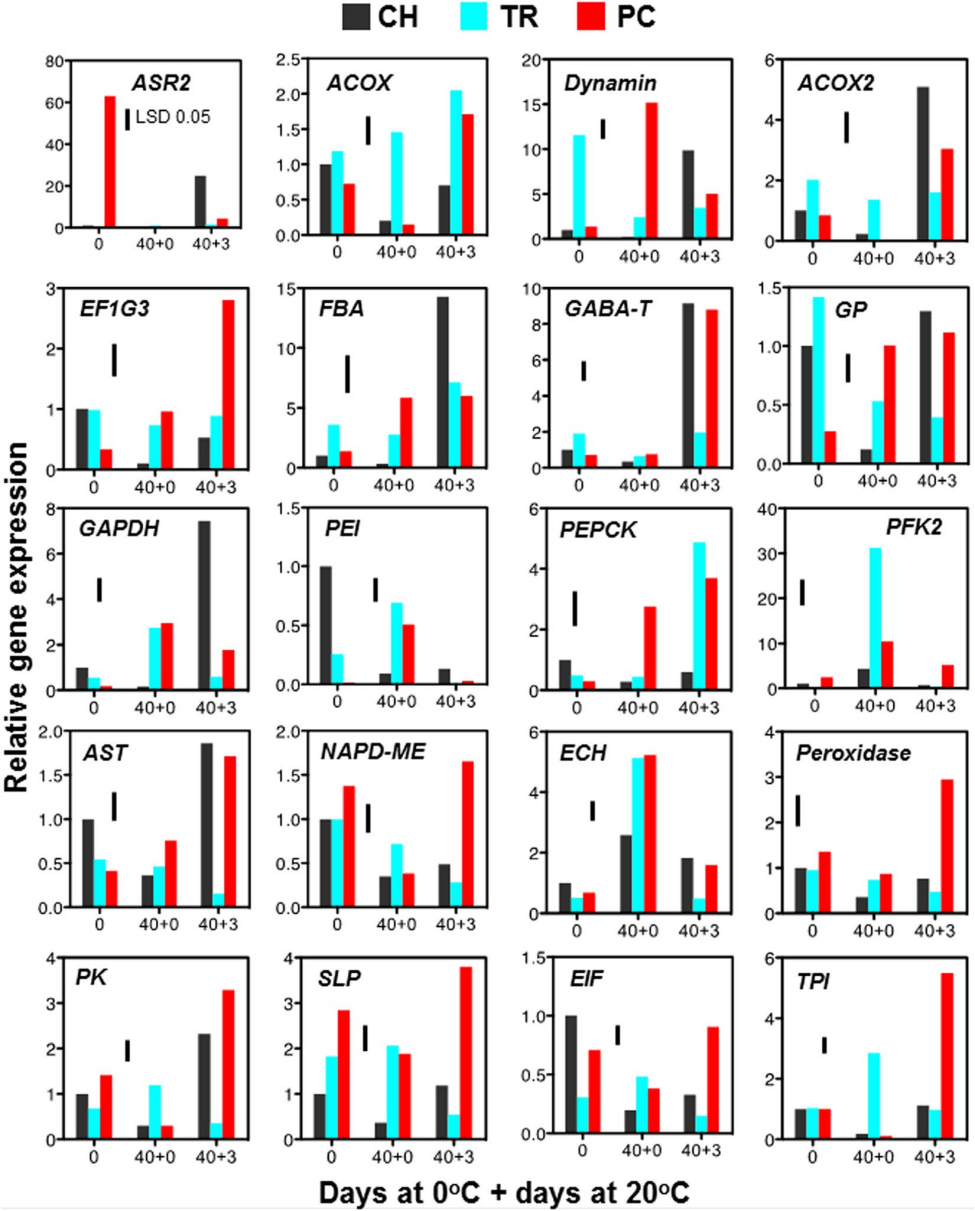
Analysis of gene expression in pre-cold and CI-symptomatic peach fruits. Analysis was performed on mesocarp tissue sampled immediately after harvest and/or before cold treatment (0 d), just after 40 d cold (40 + 0 d) and after three days ripening at 20 °C following 40 d cold treatment (40 + 3 d). Additional experimental details were as described (Fig. 1). The vertical bar in each particular figure plate represents the least significant difference (LSD, P ≤ 0.05), which was used for means comparison among treatments and time points. Gene name abbreviations: *ASR2: abscisic acid stress ripening protein homolog; ACOX: acyl-coenzyme A oxidase; ACOX2: acyl-CoA oxidase 2; EF1G3: elongation factor 1-gamma 3 isoform; FBA: fructose-bisphosphate aldolase; GABA-T: gamma-aminobutyrate transaminase; GP: glutathione peroxidase; GAPDH: glyceraldehyde-3-phosphate dehydrogenase; PEI: pectinesterase inhibitor; PEPCK: phosphoenolpyruvate carboxykinase; PFK2: phosphofructokinase beta subunit; AST: aspartate aminotransferase; NADP-ME: cytosolic NADP-malic protein; ECH: enoyl-CoA hydratase; PK: pyruvate kinase; SLP: subtilisin-like protease; EIF: eukaryotic translation initiation factor;* and *TPI: triosephosphate isomerase*.

Proteomic analysis data were further validated to examine whether the protein accumulation patterns of 20 selected proteins were matched at the transcript level. Quantitative RT-PCR was performed using RNA samples from three biological replicates of fruit prior to cold treatment (0 d), after cold treatment (40 + 0 d) and after three days ripening at 20 °C (40 + 3 d) (Fig. 7). All genes analyzed (encoded proteins listed in Supplementary Table S4) were affected by the postharvest treatments (Fig. 4). As a rule, gene expression patterns before cold treatment of *PFK2: phosphofructokinase beta subunit*; *GABA-T: gamma aminobutyrate transaminase*; *EF1G3: elongation factor 1-gamma 3 isoform*; *GP: glutathione peroxidase*; *FBA: fructose-bisphosphate aldolase*; *GAPDH: glyceraldehyde-3-phosphate dehydrogenase*; *NADP-ME: NADP-dependent malic enzyme*; *ACOX: acyl-coenzyme A oxidase;* and *TPI: triosephosphate isomerase* coincided with modifications in protein abundance (Fig. 4). The same correspondence occurred during post-cold ripening (40 + 3 d) for *PEI: pectinesterase inhibitor*; *ECH: enoyl-CoA hydratase*; *FBA: fructose-bisphosphate aldolase*; and *TPI: triosephosphate isomerase*. The expression pattern of several genes at 40 + 0 d (*e.g*. *aspartate aminotransferase*; Fig. 7) matches the corresponding protein changes at 40 + 3 d, showing that gene expression precedes protein modulation (Figs 4 and 7). Genes that were differently expressed between TR and PC fruit during the CI symptomatic period (40 + 3d), such as *PFK2; PK: pyruvate kinase; GP; SLP: subtilisin-like protease; NADP-ME; EIF: eukaryotic translation initiation factor;* and *TPI* (Fig. 7), may correlate with the phenotypes (Fig. 1A). Gene expression analysis revealed that most of the examined genes were up-regulated at post-cold stage in CI-free PC fruit. However, the majority (*n = *18) exhibited the least expression in severely CI-affected TR fruit at the same period. Meanwhile, 16 genes had low expression in CH fruits at post-cold time (Fig. 7).

### Metabolic profiling and targeted gene expression analysis

To further explore the CI responses of peach fruit, we used GC-ToF-MS analysis to monitor changes in primary metabolites in CH, TR and PC peach mesocarp tissue before and after cold treatment. The full set of primary metabolites was first subjected to Principal Component Analysis (PCA) for an overview of data dispersion across the different treatments. Globally, the data are relatively sparse in the multi-dimensional space (the first three principal components capture only 65% of the total variance), the first principal component (35% of variance) clearly separates cold-stored fruits (CH 40 + 3, TR 40 + 3 and PC 40 + 3) from the initial conditions (CH, TR and PC). The second principal component (20% of variance) roughly discriminates the TR and PC treatments (Supplementary Table S5).

**Figure 8:**
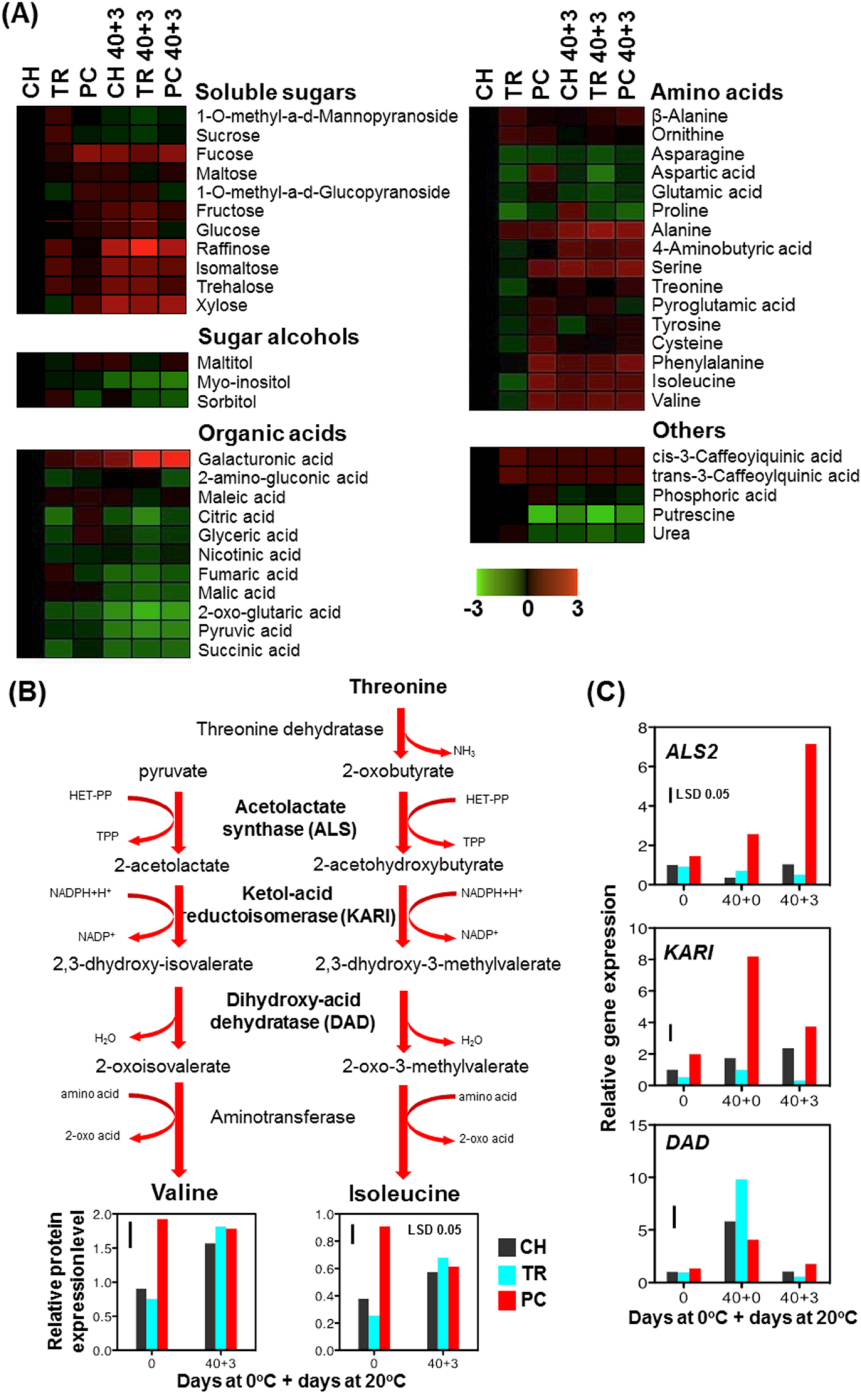
Metabolic differences and transcriptional regulation of valine and isoleucine biosynthesis in pre-cold and CI-symptomatic period. (**A**) Heat map representing relative abundance of primary metabolites analyzed prior to cold period (CH, TR, PC) and after three days ripening at 20 °C following 40 d cold treatment (CH 40 + 3, TR 40 + 3, PC 40 + 3) compared to CH. Additional experimental details were as described (Fig. 1). Normalized values are shown on a colour scale at the bottom of the figure, which is proportional to the concentration of each metabolite. Numerical data are provided (Supplementary Table S6). (**B**) Superpathway of valine and isoleucine biosynthesis in plants. Valine and isoleucine metabolite profiles for peach fruit prior to chilling and at the CI-symptomatic stage are shown (see also Fig. 8 and Supplementary Table S6). (**C**) Expression profiles for *acetolactate synthase* (*ALS2*), *ketol-acid reductoisomerase* (*KARI*) and *dihydroxy-acid dehydratase* (*DAD*) in peach fruit. Mesocarp tissue sampled immediately after harvest and before cold treatment (0), just after 40 d cold (40 + 0) and after three days ripening at 20 °C following 40 d cold treatment (40 + 3). Additional experimental details were as described (Fig. 1). Determinations were performed in triplicate. The vertical bar in each particular figure plate represents the least significant difference (LSD, *P* = 0.05), which was used for means comparison among treatments and time points.

During the CI symptomatic period (40 + 3 d), the concentrations of most soluble sugars, including fucose, fructose, raffinose, isomaltose, trehalose, and xylose, increased (highlighted with red color) in comparison to before chilling, while concentrations of organic acids decreased (highlighted with green color), with the exception of galacturonic acid that increased strongly (Fig. 8A; numeric data are provided in Supplementary Table S6). Interestingly, most amino acids increased, including alanine, 4-aminobutyric acid, serine, phenylalanine, isoleucine and valine; however, several other nitrogen-containing compounds, such as aspartic acid, putrescine and urea, were less abundant during the CI symptomatic period than before chilling (Fig. 8A; Supplementary Table S6).

Comparing the metabolic profiles of peach fruit before chilling and when expressing CI symptoms (40 + 3 d), less noticeable changes were observed in CI-free PC fruit than in severely CI-affected TR fruit (Fig. 8A; Supplementary Table S6). For instance, 24 metabolites showed conspicuous changes in TR, 11 in CH and only nine in PC fruit during the CI symptomatic period than before chilling (Fig. 8A; Supplementary Table S6). As expected, fruits with contrasting CI phenotypes (TR 40 + 3 d *versus* PC 40 + 3 d) also had different concentrations of some metabolites (*n* = 30), including all soluble sugars, the majority of amino acids [alanine, serine, threonine, beta-alanine, 4-aminobutyric acid (GABA), aspartic acid, pyroglutamic acid, phenylalanine, and asparagine], organic acids (glyceric acid, fumaric acid, maleic acid, nicotinic acid, citric acid, galacturonic acid, and 2-amino-2-deoxy-D-gluconic acid), maltitol, phosphoric acid and urea (Fig. 8A; Supplementary Table S6). A more prominent source of metabolic variation was noted before cold treatment: TR and PC fruits differed in 30 metabolites. Pre-conditioned fruits that showed a CI-free phenotype had higher concentrations of several amino acids (aspartic acid, glutamic acid, serine, pyroglutamic acid, tyrosine, cysteine, isoleucine, valine, threonine, and phenylalanine), sugars (fucose, 1-O-methyl-alpha-D-glucopyranoside, maltose, and xylose), sugar-alcohols (maltitol) and organic acids (glyceric acid and citric acid) than TR fruits that eventually expressed severe CI symptoms, even prior to cold (Fig. 8A; Table Supplementary S6).

Among amino acids with significantly different concentrations in TR and PC fruit prior to cold exposure were valine and isoleucine (Fig. 8A; Supplementary Table S6), which share four steps of their biosynthetic pathways as part of the super-pathway of valine, isoleucine and leucine biosynthesis in plants (Fig. 8B). This interesting observation led us to analyze expression of three key genes of this pathway by qRT-PCR to assess transcriptional regulation of valine and isoleucine biosynthesis in fruits with contrasting CI responses and further understand any potential roles for these amino acids in CI development and/or tolerance. During ripening after cold treatment (40 + 3 d), the greatest expression of acetolactate synthase (*ALS*), ketol-acid reductoisomerase (*KARI*) and dihydroxy-acid dehydratase (*DAD*) were in PC fruit, followed by CH and TR fruit. There was strong up-regulation of *ALS* and particularly of *KARI*, just after cold treatment (40 + 0 d) and prior to when CI symptoms develop (40 + 3 d), in CI-free PC fruit compared to the eventually CI-affected TR fruit (Fig. 8C). Overall, this targeted analysis effectively highlights metabolic pathways actively involved in CI responses.

## Discussion

Chilling injury is the major disorder of several fleshy fruits, including peach^4, 6, 11, 14, 17^; however, how CI development is regulated is not fully understood. One way to address the mechanism of CI tolerance/sensitivity is to analyze cellular changes associated with treatments that avoid or decrease CI symptoms^7, 8^. A comparative protocol of controlled peach fruit ripening that included harvest at commercial stage (CH), off-tree ripening for two days at room temperature (PC) and on-tree ripening to the same degree (TR) was used to evaluate CI development (Fig. 1). Phenotypic data indicated that PC fruit exhibited a CI-free phenotype, TR fruit was severely CI-damaged, and CH fruit showed an intermediate phenotype after 40 d cold plus three days ripening at 20 **°**C (Fig. 1). These observations were further validated by measurements of CI-related downstream events, such as expressible juice, anthocyanin pigments in mesocarp tissue, mealiness, and browning and bleeding indices (Fig. 2C–I). These data, together with previous findings^15^, suggest that pre-conditioning treatments serve as an efficient experimental system to characterize priming-like mechanisms against cold stress.

It is noted that TR fruit had a higher ripening index than PC at harvest and after cold treatment (Fig. 2C and Supplementary Fig. S1), however no difference in mesocarp firmness was found (Fig. 2B). This result is in accordance with previous studies^10, 14^, while others report that CI is accompanied by greater fruit firmness^18^. TR-damaged fruit exhibited reduced respiration after cold treatment (Fig. 2A; Supplementary Fig. S2), consistent previous results^19^.

Ethylene synthesis, perception and signal transduction is essential to coordinate climacteric ripening^20^. Involvement of ethylene in fruit cold responses has been reported previously^2^, although a clear relationship between ethylene synthesis and CI remains to be established^6^. Ethylene biosynthesis data indicated that CI-free PC fruit had increased steady-state ethylene, ACC and MACC concentrations and ACS and ACO enzymatic activities than the CI-affected TR fruit (Fig. 3). This suggests that fruit pre-conditioning maintains an active ethylene biosynthesis pathway, enabling normal fruit ripening after cold.

To specifically explore this phenomenon, we deployed proteomic analysis to compare samples before chilling and during the period where CI symptoms develop to identify proteins associated with different degrees of CI. After cold treatment, healthy fruits maintained higher levels of accumulation for several (*n* = 12) proteins involved in energy, metabolism, protein destination/storage, defence and signal transduction than CI-affected fruit. This is consistent with previous findings of a general repression in gene expression in CI-affected peach fruit^21^. Among these PC-induced proteins, several enzymes related to energy metabolism, including triose phosphate isomerase, NADP-dependent malic enzyme and glyceraldehyde-3-phosphate dehydrogenase, were identified. The down-regulation of the corresponding genes after cold treatment in TR fruit (Fig. 7), along with decreased protein abundance of triosephosphate isomerase and NADP-dependent malic enzyme (Fig. 4), may account for the decreased respiration (Fig. 2A) and could contribute to CI symptoms development due to decreased energy production^17^. After chilling, two proteins associated with sugar/lipid metabolism, phosphoenolpyruvate carboxykinase [ATP] and enoyl-CoA hydratase, were increased by PC over amounts in TR fruits (Fig. 4), concomitant with up-regulation of *PEPCK* and *ECH* expression (Fig. 7). As phosphoenolpyruvate carboxykinase activates the gluconeogenesis pathway from organic acids to sugars in tomato^22^ and gluconeogenesis from malate occurs in peach during ripening^23^, it is possible that this pathway contributes to the observed normal, ethylene-driven ripening seen in PC fruit (Fig. 3). The inability of TR fruit to ripen normally after cold is further documented here with the decreased abundance of endopolygalacturonase (Fig. 4), a well-studied enzyme that degrades pectin and provokes cell wall loosening in peach fruit during ripening^24^. Another protein that ensures normal fruit ripening is pectinesterase inhibitor, a regulator of pectinesterase activity^25^. In our study, both accumulation of pectinesterase inhibitor and expression of its encoding gene (*PEI*) were commonly modulated by PC and TR treatments. Although this response does not explain the distinct CI phenotype (Fig. 1), it may reflect the similar pattern of fruit softening (Fig. 2B).

In addition to ripening, ethylene is also involved in defense signaling related to cold^26^. A striking feature revealed by our proteomic analysis is the up-regulation of several proteins associated with defense in PC compared to TR fruit following chilling (Fig. 4). Many defense-related proteins, including the major allergen ankyrin repeat domain-containing protein and HSP 70, were also previously associated with CI in peach fruit^11, 17^. Notably, stromal cell-derived factor was induced by PC before cold treatment. Although the exact role of stromal cell-derived factor in plant defense remains uncertain, this protein is likely to enforce the healing mechanism of peach fruits against CI symptoms, as suggested in injured animal cells^27^.

As indicated elsewhere^21^ and in more detail here, there is a correlation between visual CI symptoms and the abundance of abscisic acid stress ripening protein (ASR). Indeed, ASR protein accumulated in TR fruit during the CI symptomatic period (Fig. 4). This response is in line with the *ASR2* expression pattern before cold and with the appearance of CI symptoms; however, TR fruits exhibit the lowest *ASR2* expression (Fig. 8), indicating that ASR repression might enhance CI susceptibility. On the other hand, the up-regulation of both GTP binding protein dynamin and *dynamin* expression in PC fruits (Figs 4 and 7) is consistent with their known function in cell wall and plasma membrane signaling^28^. Several proteins involved in protein synthesis, such as 60 S ribosomal protein, eukaryotic translation initiation factor and elongation factor 1-gamma 3 isoform, accumulated in PC fruit after chilling (Fig. 4), confirming that protein synthesis is critical for enhanced CI acclimation^14^. This suggestion is further supported by the remarkable up-regulation of *EF1G3* and *EIF* observed after chilling in PC fruit (Fig. 8). On the other hand, flesh browning and red pigment accumulation (bleeding) are commonly associated with CI phenotype in peach fruit^5, 6^. A particularly intriguing observation here was the greater accumulation of leucoanthocyanidin dioxygenase in TR than in PC fruits after chilling (Fig. 4). Because this enzyme participates in anthocyanidin biosynthesis^29^, we anticipate that this pathway may be involved in CI-associated internal flesh bleeding and/or browning in peach fruit (Fig. 1).

To ascertain whether the individual treatments directly affected ripening and subsequent cold responses, we also analyzed protein signatures before cold. Notably, there were differences in 60 proteins between TR and PC fruit before chilling (Fig. 4), much more than the 16 proteins that were different after chilling, showing that the ripening history before cold induced cellular modifications that might specifically alter chilling-responsive regulation in peach fruit. Of particular interest, overlaps between PC and TR only occurred for leucoanthocyanidin dioxygenase and stromal cell-derived factor, of which the first was down-regulated and the second, up-regulated in PC fruit compared to TR, both before and after chilling (Fig. 4). This finding further enforces the key role of leucoanthocyanidin dioxygenase as an early marker for CI-associated flesh-bleeding symptom in peach.

Previous studies proposed a crucial role played by peroxisomal *β*-oxidation of saturated fatty acids in postharvest peach ripening and suggested that lactone biosynthesis promotes CI acclimation^30^. Our data also showed that prior to chilling, PC fruit accumulated more of two acyl-CoA oxidase isoforms that catalyze the first and rate-determining step of peroxisomal *β*-oxidation of fatty acids (Fig. 4). Increased activity of acyl-CoA oxidase is linked to biosynthesis of lactones, peach-like aroma volatiles, maintaining aroma quality in CI-free, but not CI-affected, peaches^30^. Thus, it is possible that controlled delayed cooling (pre-conditioning) alleviates CI development by stimulating acyl-CoA oxidase and lactone production. Furthermore, the greater abundance of acyl-CoA oxidase in the PC treatment (Fig. 4) could be attributed to enhanced ethylene production (Fig. 3E), because volatile lactone production via regulation of acyl-CoA oxidases is an ethylene-dependent ripening process in peach^31^. Moreover, the absence of correlation between the abundance of acyl-CoA oxidase proteins and the expression pattern of *ACOX* and *ACXO2* has also been reported previously^30^.

Considerable differences in the representation of some functional categories were also evident between TR and PC fruit before chilling. The strong impact of amino acid metabolism on CI phenotype is illustrated by differences in several proteins, including aspartate aminotransferase, gamma-aminobutyrate transaminase and NAD-dependent malate dehydrogenase, in transcripts encoding these components, including *AST* and *GABA-T*, and in several metabolites, like aspartic acid, glutamic acid, and GABA among the different CI phenotypes. Particularly, the reduced concentration of GABA in TR compared to PC fruit prior to cold treatment suggests that GABA may be involved in CI tolerance. Such a mechanism is plausible, as exogenous application of GABA alleviated CI in banana^32^.

Comparative profiling of primary peach fruit metabolites revealed clear differences in the accumulation of several metabolites between fruits before and after chilling. Among major groups of metabolites with significant differences between TR and PC before chilling were sugars (fucose, maltose and xylose), sugar alcohols (maltitol and sorbitol), organic acids (glyceric acid and citric acid), amino acids (aspartic acid, serine, tyrosine, cysteine, phenylalanine, isoleucine and valine) and nitrogen-containing compounds (putrescine and urea; Fig. 8). The majority of these metabolic differences between TR and PC fruits were unaffected by chilling, while several new metabolic differences occurred during the period when CI symptoms develop (1-O-methyl-a-D-glucopyranoside, glucose, 2-amino-gluconic acid, maleic acid and pyroglutamic acid), but were not different before chilling (Fig. 8). Thus, different CI phenotypes that develop after chilling may reflect differential metabolic reconfigurations occurring during chilling, suggesting that CI responses can be sensed differently depending on the pre-chilling history of the peach fruit. The greater accumulation of valine, isoleucine, proline, serine, threonine, aspartic acid, cysteine, pyroglutamic acid, glutamic acid, phenylalanine and tyrosine in PC than in TR fruit before chilling indicated that specific sectors of primary metabolism are altered (Fig. 8). These changes resemble metabolic priming associated with cold acclimation^33^ and indicate that changes associated with the amino acid pool size alter peach metabolism and perhaps CI responses.

Although some changes in CI-affected metabolites reported here (raffinose, aspartic acid, threonine, tyrosine, cysteine, phenylalanine and putrescine) are similar to those found in a previous peach CI study^12^, several CI-associated metabolic responses of ‘June Gold’ peaches found here were not observed in other peach genotypes^12, 13^. It was suggested that raffinose accumulation after chilling may serve as a biomarker of CI resistance in six different peach varieties^13^. Our data do not support these findings because the raffinose concentration (Fig. 8) did not correlated with CI development (Fig. 1), confirming that peach cultivars exhibit wide phenotypic and metabolic diversity in their response to CI^17^.

Proteomic and transcriptomic analyses indicated that specific sectors of peach fruit metabolism were different in CI-affected and healthy fruits. In particular, PC treatment before chilling repressed fructose bisphosphate aldolase and pyruvate kinase (Fig. 5), accompanied by *FBA* down-regulation (Fig. 7), providing evidence for the importance of the glycolytic pathway in CI regulation. These findings may be associated with the increased fructose and glucose found after chilling (Fig. 8), which in turn may explain the decreased sucrose concentration in PC fruit before chilling (Fig. 8; ref. 34), verifying that changes in sugar metabolism are associated with CI development^12^. This hypothesis is also supported by the distinct accumulation of phosphofructokinase beta subunit (Fig. 4) and *PFK2* expression (Fig. 7) observed in cold-stored fruit exposed to PC compared with TR. As most of these changes precede any measurable CI symptoms, it is unclear whether these sugar alterations in peach fruit are causally related to cold acclimation or whether they act indirectly as an energy and carbon source for subsequent metabolic changes leading to enhanced CI acclimation, as in *Arabidopsis* ^35^.

One of the most apparent metabolic signatures that emerged from this work is the remarkable increase in concentrations of several amino acids, including isoleucine and valine, in PC fruit prior to chilling (Fig. 8A). A similar increase in concentrations of isoleucine and valine after three days of ripening following harvest was found previously in peach fruit^12^. This suggested that accumulation of these amino acids could serve as a priming strategy to protect peach fruits from CI damage induced by subsequent chilling stress. Because both isoleucine and valine are components of the same superpathway of valine, isoleucine and leucine biosynthesis (Fig. 8B), the valine and isoleucine biosynthesis genes were examined to understand the importance of this pathway in CI expression. Consistent with isoleucine and valine accumulation just after postharvest treatments, this analysis revealed that *ALS* and particularly *KARI* were already up-regulated in PC compared with TR fruits prior to the appearance of CI symptoms, suggesting a prominent role of these branched-chain amino acids in CI development (Fig. 8C). This finding concurs with previous reports that valine and particularly isoleucine confer a cold resistance phenotype on *B. subtilis* ^36^. Since most of the labeled isoleucine was retained within the membrane fraction^36^, it is possible isoleucine to be a necessary precursor for cold-stressed peach cells for the adaptation of the fatty acid profile to adjust membrane fluidity. Alternatively, the accumulation of isoleucine and valine may serve as substrates for the synthesis of stress-induced proteins and these branched-chain amino acids may act as signaling molecules to regulate gene expression^37^. In support, a set of genes encoding proteins that are rich in branched-chain amino acids has been induced by cold stress in barley^38^. Another as yet untested hypothesis is that isoleucine and valine could be critical for protein structure and function, for instance under cold-stress conditions because of their unsubstituted aliphatic side chains with branched alkyl groups. The branched-chain amino acids are the most hydrophobic among the standard protein amino acids; isoleucine and valine are frequently located in the protein core and play a central role in determining protein structure and interaction of the transmembrane domains of membrane proteins with phospholipid bilayers^37^. The exact role of these two amino acids in CI regulation of peach fruit, once fully explored, may illuminate the mechanism of CI symptom development.

### Conclusions and future perspective

Cold responses of peach fruit are markedly altered by previous post-harvest handling. These alterations are accompanied by corresponding changes in the patterns of genes, proteins and metabolites, and in pre-conditioned fruits, resulting in cold acclimation in terms of CI expression signatures. The data reported here, in combination with previous findings, will expand our understanding of cold-responsive metabolic pathways and facilitate our ability to predict how fruit will respond to cold stress.

## Methods

### Fruit material, experimental approach and sampling procedure

‘June Gold’ peaches, a melting flesh (MF) cultivar, were grown in 2012 under standard agricultural practices in a commercial orchard at Velventos, Kozani, in northern Greece. Peach fruits were harvested either at the commercial harvest stage (CH; 97 days after full bloom; mesocarp flesh firmness: 45.3 N) or after three days at the tree ripe (TR) stage (flesh firmness: 23.8 N). Immediately after harvest, CH and TR fruit were exposed to cold treatment conditions (0 °C, RH 95%). Additionally, a group of CH fruits were maintained at 20 °C (RH 90%) for two days after harvest before cold treatment (pre-conditioning, PC) to reach the same flesh firmness (23.7 N) as TR fruit. Subsequently, the three groups of peaches (CH, TR and PC) were ripened three days at 20 °C (RH 90%) following up to 40 days cold treatment (0 °C, RH 95%). Peach samples (three replicates of five fruits each) were taken prior to cold treatment (‘pre-cold period’), immediately after cold (‘cold period’ or ‘40 + 0 d’) and after a three-day ripening period following 40 days cold treatment (‘CI-symptomatic period’ or ‘40 + 3 d’) and characterized physiologically and evaluated for CI symptoms. Mesocarp flesh samples were collected from all fruits, flash frozen in liquid nitrogen and stored at −80 °C. Samples were also taken after 20 days cold treatment (0 °C, RH 95%) for physiological analysis (Supplementary Figs S1 and S2). A schematic representation of the experimental design and fruit sampling is provided (Fig. 1).

### Fruit physiological analysis

Peach mesocarp flesh firmness was measured as Newtons (N) using a fruit texture analyzer (model 53205, T.R. Turoni srl, Forlì, Italy), as described (Karagiannis *et al*., 2016). The juice SSC was evaluated by digital refractometer (Atago PR-1, Atago Co Ltd., Tokyo, Japan)^39^.

Statistical analysis was performed using SPSS 19.0 for Mac OS X (SPSS, Chicago, IL, USA). Values (three replicates per treatment, each consisting of five fruits) were subjected to analysis of variance and least significant differences (LSD) at the 5% level were used to compare means.

### CI symptom evaluation

Chilling injury symptoms, such as mealiness, browning and bleeding were evaluated subjectively in peach fruit mesocarp immediately after the fruits were cut in half. Mesocarp tissue mealiness was scored using an arbitrary three-point reference scale where 0: juicy fruit, 1: moderately mealy fruit (small amount of juice released upon squeezing) and 2: severely mealy fruit (almost no juice released upon squeezing). Internal bleeding was visually recorded as the presence or absence of red pigmentation diffusion into the mesocarp using a scale where 0: sound fruit with no browning or bleeding; 1: moderate browning or bleeding covering one to 50% of the flesh; and 2: severe browning or bleeding covering most of the flesh (51 to 100%)^4^. For subjectively estimated CI symptoms (mealiness, bleeding, browning), an index was used to express a single grade for each symptom for each replicate using the following formula: Index = (number of fruit given score 2 × 1.0) + (number of fruit given score 1 × 0.5) + (number of fruit given score 0 × 0)/total number of fruit evaluated. Fifteen fruit of each treatment were examined at each observation time. Expressible juice and anthocyanin concentration were measured to further describe mealiness and internal bleeding, respectively. The amount of expressible juice was determined according to ref. 5. Total anthocyanin concentration was estimated using a previously described extraction and quantification method^40^.

### Ethylene production and respiration rate

Ethylene production was determined by gas chromatograph (GC; model 3300, Varian Analytical Instruments, CA, USA) analysis^20^. Respiration rate (RR) were determined with infrared gas analyzer (Combo 280, David Bishop Instruments, UK)^20^. For each treatment, three replicates of three fruits were used. Statistical analysis was performed as described above.

### Metabolites and enzymes of ethylene biosynthetic pathway

The concentrations of aminocyclopropane-1-carboxylic acid (ACC) and 1-malonyl-aminocyclopropane-l-carboxylic acid (MACC) and enzymatic activities of ACC synthase (ACS) and ACC oxidase (ACO) were analyzed using GC^41^. Statistical analysis of data was performed as described above using three biological replicates per treatment.

### Two-dimensional gel electrophoresis (2DE-PAGE), nanoLC-MS/MS analysis and data analysis

Mesocarp tissue from before-chilling (CH, TR, and PC) and symptomatic (CH 40 + 3, TR 40 + 3, and PC 40 + 3) samples was ground in liquid nitrogen and soluble proteins were extracted as previously described^42^. Protein concentrations were determined following Bradford’s method (Bradford, 1976). Fifty micrograms (50 μg) of soluble proteins were analyzed by 2DE-PAGE according to^43^. 2DE-gels were stained with silver nitrate, scanned with a Bio-Rad GS-800 calibrated densitometer and analyzed with the PDQuest advanced 2-D gel analysis software^44^. For each treatment, 2DE-maps were run in triplicate and for a minimum of three independent extractions each correspond to a biological replication. Statistical analysis was done by one-way ANOVA (with significance at P = 0.05) and individual means were compared using Student’s t-test (significance of 95%). The statistically significant differences were further combined by a quantitative 1.5-fold change of spot volume.

Selected spots of interest from gels run in parallel and stained with the Silver Stain Plus kit (Biorad) were analyzed by LTQ-Velos-Orbitrap (Thermo Fisher Scientific, Bremen, Germany) online with a nanoliquid chromatography (LC) Ultimate 3000 system (Dionex, Sunnyvale, CA, USA). For protein identification, tandem mass spectrometry (MS/MS) experiments were performed as reported^45^. For heat diagram preparation, the mean of each spot volume from the various treatments was expressed as a ratio of the mean spot volume at CH and then the ratio was log2-transformed.

### Gene expression analysis

NA was isolated from fruits just after harvest (CH, TR, and PC), after cold treatment (CH 40 + 0, TR 40 + 0, and PC 40 + 0) and after CI symptoms developed (CH 40 + 3, TR 40 + 3, and PC 40 + 3) using the RNeasy plant RNA isolation kit (Qiagen, Crawley, UK). RT-PCR was performed according to ref. 46 Primer assays for the selected 20 genes derived from proteomic analysis (*ASR1, ACOX, ACX2, DNM1, EF1, FBA, GABA-T, GSH, GAPDH, PTEN, PEPCK, PFP-beta, ASAT, NADP-ME, ECH, PRX, Kin, SUB, eIF*, and *TPI*) and three genes derived from metabolomic analysis (ALS2, DHAD and KARI) were designed with Primer3Plus (http://www.bioinformatics.nl/cgi-bin/primer3plus/primer3plus.cgi) (Supplementary Table S1). Additionally, specificity of the assays was tested in silico using blast (http://blast.ncbi.nlm.nih.gov/Blast.cgi). The qPCR was performed according to ref. 47 The cycling program was: two min at 95 °C to activate the polymerase, followed by 35 cycles of denaturation at 95 °C for 10 s, annealing at 55 to 62 °C for 15 s and elongation at 72 °C for 20 s. Post-PCR melting curves were measured from 65 to 95 °C in 0.5 °C intervals to validate the formation of expected PCR products. Data were analyzed with GenEx (MultiD, version 6.1). Off-scale data were removed during pre-processing using a cutoff at 36 cycles and outliers were identified with Grubb’s test. All data were normalized to the spike and converted to relative quantities (relative to the highest Ct for each gene, after arbitrarily assigning an expression of one to the least-expressed sample). The last step of pre-processing was to transform the data to log2 scale. Three biological replicates of each treatment were performed and used for the gene expression experiments.

### Analysis of metabolites by gas chromatography–time of flight–mass spectrometry (GC-ToF-MS)

At the same times sampled for proteomic analysis, primary metabolites were also extracted from 250 mg frozen mesocarp powder, derivatized and analyzed using GC-ToF-MS according to established procedures^48^. Peaks were identified using Tagfinder^49^ and their identity confirmed using the mass spectral tags in the MPI-MP Golm database^50^. Relative amounts of metabolites were normalized on the peak area of the internal standard ribitol as described^51^. Principal component analysis (PCA) was calculated based on metabolite correlations starting from the normalized datasets obtained from GC-MS profiling. The normalized values of the 46 metabolites were also analyzed by one-way ANOVA followed by the LSD test to detect significant differences (*P* = 0.05). Further details regarding the protocols for metabolic profiling and the full data set of identified metabolites are reported in Supplementary Table S2 following established guidelines^52^. A heat diagram of metabolite changes was prepared as described above for proteomic data. Mean values of six independent determinations for each treatment and time point were expressed as the log2-transformed ratio between each stage and CH using the Multi Experiment Viewer software (MeV v4.4.1; ref. 53).

## Electronic supplementary material

Supplementary Dataset 1

Supplementary Table S2

Supplementary Table S3

Supplementary Table S4
