# Supplementary material for: Exploring priming responses involved in peach fruit acclimation to cold stress
Source: Sci Rep. 2017 Sep 12;7:11358. doi: 10.1038/s41598-017-11933-3 (PMC5595836; doi:10.1038/s41598-017-11933-3)
Supplement: Supplementary file 1 — Supplementary Dataset 1 [file 41598_2017_11933_MOESM1_ESM.pdf]

Exploring priming responses *involved in* peach fruit acclimation to cold stress

Georgia Tanou, Ioannis S. Minas , Federico Scossa, Maya Belghazi, Aliki Xanthopoulou, Ioannis Ganopoulos, Panagiotis Madesis, Alisdair Fernie & Athanassios Molassiotis

Supplementary data

Supplementary Figures 1-4

Supplementary Tables S1, S5 and S6

Additional Information

Fig. S1 Peach fruit quality and CI parameters following 20 d cold treatment.

Fig. S2 Ethylene biosynthesis during peach ripening at 20 °C following 20 d cold treatment.

Fig. S3 Reference 2DE-gel map of peach mesocarp tissue.

Fig. S4 Representative 2-DE gels exhibiting protein abundance changes for pre-cold and symptomatic fruit.

Fig. S5 VENN diagram displaying the effect of cold and ripening in each treatment.

Table S1 Primers used in this study.

Table S2 Metabolite reporting guidelines.

Table S3 Quantitative data for peach protein spot volumes.

Table S4 Sequence data for peach proteins.

Table S5. Principal component analysis of metabolic profiles.

Table S6. Quantitative results of metabolite analysis.

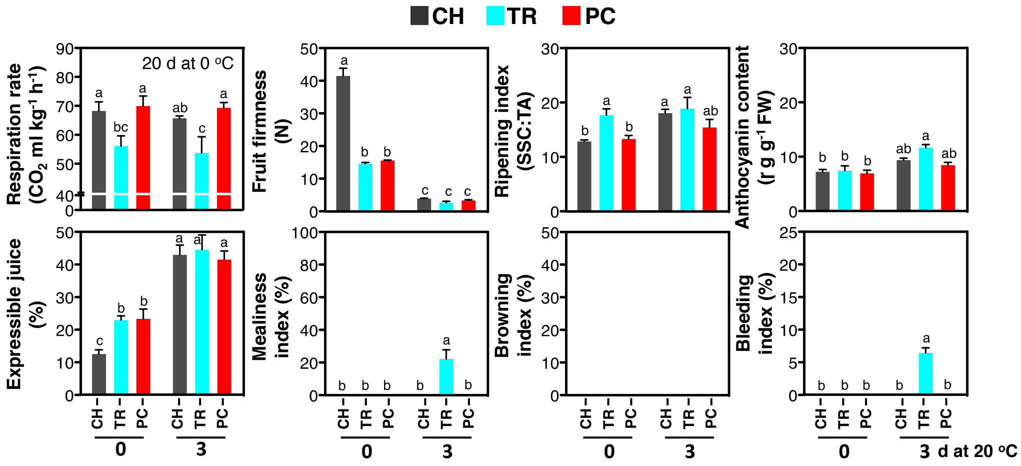

Supplementary Figure 1

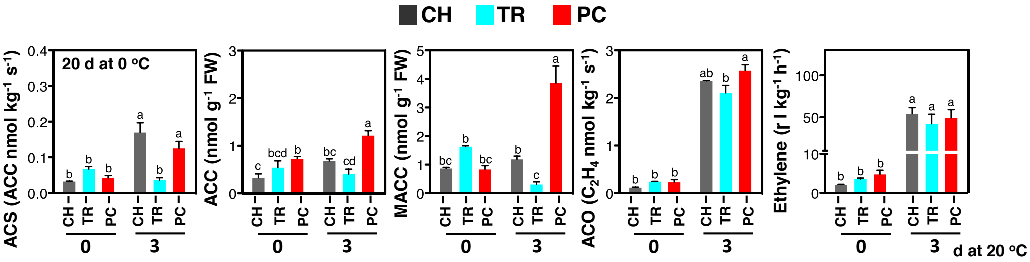

Supplementary Figure 2

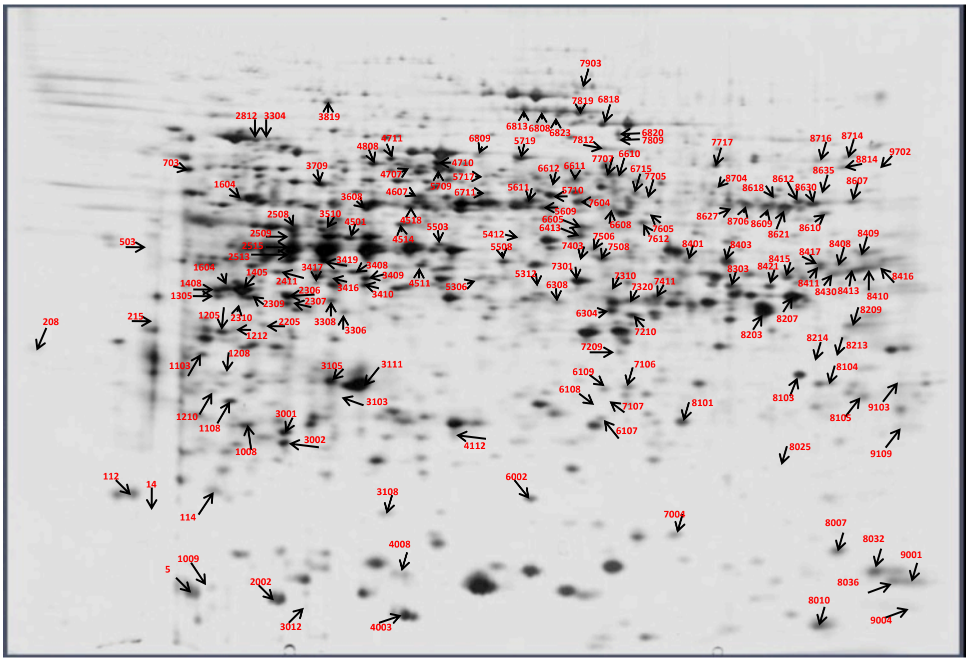

Supplementary Figure 3

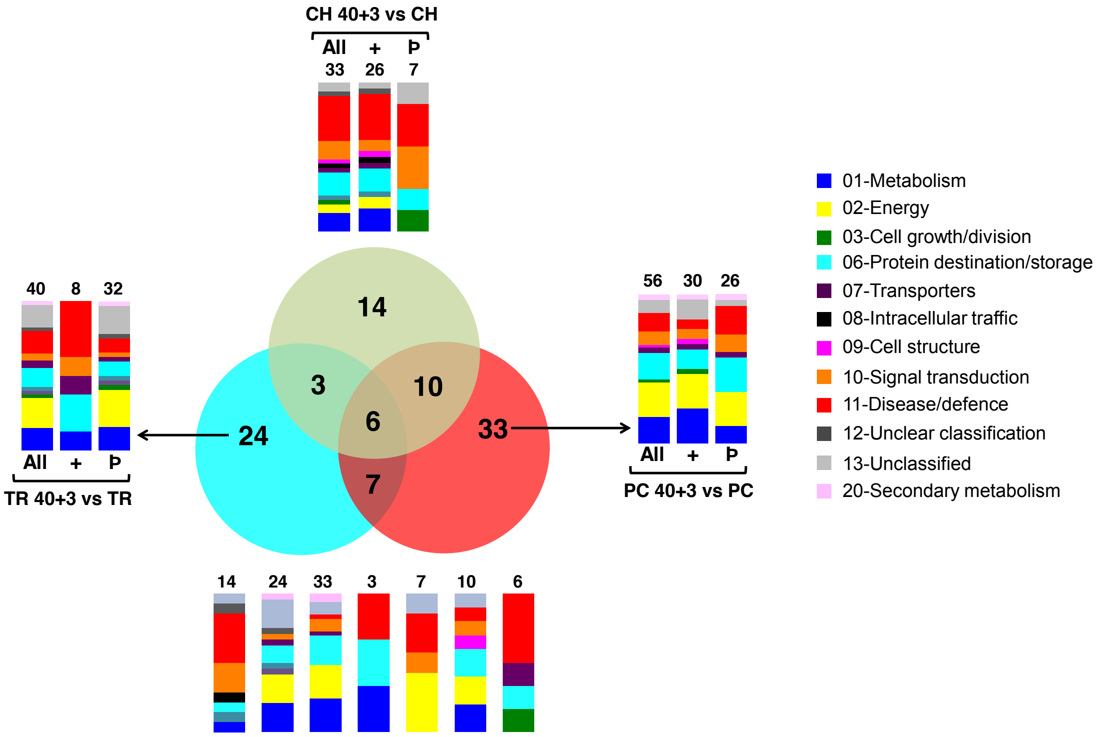

Supplementary Figure 4

Supplementary Table S1: Primers used for quantitative real-time PCR assays.

**Primer name**	**Forward 5’-3’**	**Reverse 5’-3’**	**Gene /enzyme target**
Ppe ASR1	GGCCTCTTCAACCAGGACAGAG	CTAGCACGACGCTCTTCACCAC	ASR1
Ppe ACOX	ATGGAGGGCGTTGATCATTTAGC	ATCCGTTTCCATCCATGAGCTG	ACOX
PpeACX2	ATGCAAAGCCCCTACCCAGATC	CGTCTGAAGATCAGGCCTGTTG	ACX2
PpeDynam	GGAGGCGATCGAGGAGTTGTTG	TAATGGGAGCCCGAGTAGCACC	DNM1
PpeEF	GTCGCAACAAAAACCCTAGCCAGC	GTCTTCCCTGCATGCAAGACCAG	EF1
PpeFBA	ATGTCGGCCTTCAAGGGAAAGTAC	CAGCTGTCTTTTGGTAGAGGGTTTC	FBA
PpeGABA-T	GTCTTCTTGGATCCAACCTCAGAAC	AGCATCTCATGCCCCTTGAACC	GABA-T
PpeGSH	GCTCTGCCCTTCTACTCGCTTTC	CGATCGCAAAGTACACCAAACCGG	GSH
PpeGAPDH	GGGGAAGATCAAGATCGGAATCAAC	TGTTTGCGTCCCTAAGCTTGACATC	GAPDH
PpePTEN	TGAACAACTCAACATCTCTCCTTCAAC	CTTCACTATATCCACCAGTATTACGCC	PTEN
PpePEPCK	GGTGAAGGCGAAGACCATCGAC	CTTCTGGCCCTCCGATTTCCTC	PEPCK
PpePFP-beta	ACCTGGAGGGCACAATGTGATTTC	GGCCATCCAAATCCAGCTTCAC	PFP-beta
PpeASAT	AGGATCCAATACTTGGGGTGACTG	CACCAAGAATCAGCTTAGCACTCAG	ASAT
PpeNADP-ME	GAAACGGCAGCGTAGTGATGGAG	TGGTGTGGATCCCGTATCAACG	NADP-ME
PpeECH	TTATGAATGGGATTGCAATGGGAGC	TTTCAGCACCATCCAACATTGTACC	ECH
PpePRX	GGTGTGGTGGAGCAAGCTCAAC	GGGCAAACATTCTCGAGTGCAG	PRX
PpeKin	GAACGTCGCCCGCTTTAACTTC	AATGGTGATTTCCTGCCCTTCCTT	Kin
PpeSUB	AGAACATCCAGGTGGTGACTAGG	ATTCGGCGTACCCACATTTGTC	SUB
PpeeIF	CCCACAAGCTGGAGAGGAAGT	CCCTCCATTAGCACACTCAGG	eIF
PpeTPI	TCCACATCTCTCGCTTCCCAAC	CAAAGAACTTTCCAGTGCCGGC	TPI
PpeDihAcidDehyDr	CATCTAATGGGCCCCACCACCTCAG	ACTCCGTGTAGAATGGCCTGGGACC	dihydroxy-acid dehydratase
PpeketRedIso	TGTCGTCAGCATCCAAAACCCACG	GGGGGCTTAACAGTAGGCATTGACACC	NADP+
PpeAcetLacSyn	CCATTGCTCCACCTTCTTCCGCCTC	TAGTGGTGGTGATGGTGGTGGTGGC	ALS

Supplementary Table S5

Supplementary Table S5. Principal component analysis for comparison of metabolic profiles.

PC	Eigenvalue	Percent	Cumulative percent
1	16,570	35,256	35,256
2	9,376	19,949	55,205
3	4,496	9,567	64,771
4	4,251	9,044	73,815
5	3,820	8,127	81,942
6	1,945	4,139	86,081
7	1,389	2,955	89,036
8	0,947	2,015	91,051
9	0,914	1,945	92,996
10	0,674	1,434	94,430
11	0,451	0,959	95,389
12	0,417	0,887	96,276
13	0,322	0,685	96,960
14	0,281	0,599	97,559
15	0,264	0,563	98,122
16	0,192	0,409	98,530
17	0,145	0,308	98,838
18	0,109	0,232	99,071
19	0,085	0,180	99,251
20	0,069	0,146	99,397
21	0,053	0,113	99,510
22	0,047	0,100	99,609
23	0,039	0,084	99,693
24	0,037	0,078	99,771
25	0,030	0,064	99,834
26	0,023	0,049	99,883
27	0,015	0,033	99,916
28	0,011	0,023	99,939
29	0,009	0,020	99,960
30	0,007	0,014	99,974

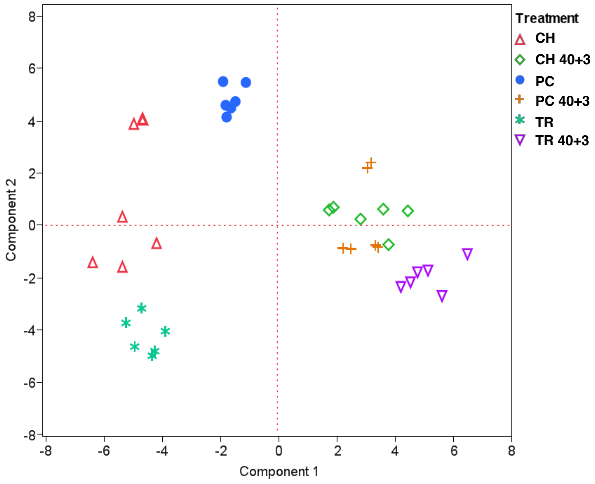

Supplementary Table S6

Supplementary Table S6. Relative metabolite levels in peach mesocarp under different treatments (expressed as normalized intensity values per g FW ± SE). Different letters indicate significant differences at the level of alpha=0.05 (LSD test)

	CH	TR	PC	CH 40+3	TR 40+3	PC 40+3
**Amino acids**						
Alanine	2.55±0.30^{d}	3.56±0.30^{cd}	3.84±0.28^{c}	6.77±0.40^{b}	9.31±0.48^{a}	7.65±0.43^{b}
Valine	0.9±0.04^{c}	0.75±0.05^{c}	1.92±0.03^{a}	1.57±0.12^{b}	1.81±0.08^{a}	1.78±0.09^{ab}
Isoleucine	0.38±0.00^{d}	0.25±0.02^{e}	0.91±0.02^{a}	0.57±0.06^{c}	0.68±0.02^{b}	0.62±0.06^{bc}
Proline	2.31±0.39^{b}	1.11±0.09^{d}	1.93±0.12^{bc}	4.06±0.4^{a}	1.67±0.27^{bcd}	1.23±0.03^{cd}
Serine	2.39±0.18^{d}	2.19±0.1^{d}	4.30±0.16^{c}	6.19±0.23^{a}	4.91±0.18^{b}	6.42±0.25^{a}
Threonine	1.30±0.08^{b}	0.96±0.06^{c}	1.33±0.07^{b}	1.47±0.04^{ab}	1.29±0.04^{b}	1.56±0.09^{a}
Beta-Alanine	0.030±0.001^{c}	0.040±0.002^{a}	0.031±0.001^{bc}	0.031±0.001^{bc}	0.034±0.002^{b}	0.039±0.000^{a}
4-aminobutyric acid (GABA)	0.84±0.03^{c}	0.76±0.03^{c}	0.85±0.02^{c}	1.43±0.10^{a}	1.16±0.10^{b}	1.46±0.08^{a}
Aspartic acid	5.47±1.35^{b}	3.47±0.09^{bc}	9.27±0.67^{a}	4.94±0.63^{b}	2.27±0.25^{c}	4.85±0.43^{b}
Cysteine	1.05±0.04^{bc}	0.88±0.07^{c}	1.66±0.08^{a}	1.10±0.02^{b}	1.10±0.05^{b}	1.17±0.07^{b}
Pyroglutamic acid	2.72±0.46^{bc}	2.56±0.08^{c}	3.40±0.18^{a}	2.95±0.08^{abc}	3.24±0.19^{ab}	2.44±0.09^{c}
Glutamic acid	2.25±0.40^{ab}	1.84±0.04^{bc}	2.48±0.14^{a}	1.86±0.10^{bc}	1.53±0.10^{c}	1.88±0.09^{bc}
Phenylalanine	0.24±0.00^{c}	0.23±0.02^{c}	0.56±0.03^{a}	0.35±0.03^{b}	0.38±0.03^{b}	0.55±0.04^{a}
Asparagine	14.52±0.19^{a}	10.69±0.63^{b}	10.35±0.49^{bc}	11.17±0.26^{b}	9.17±0.43^{c}	11.43±0.60^{b}
Ornithine	0.038±0.007^{a}	0.051±0.013^{a}	0.044±0.002^{a}	0.037±0.000^{a}	0.040±0.004^{a}	0.039±0.002^{a}
Tyrosine	0.156±0.005^{bc}	0.136±0.010^{cd}	0.198±0.006^{a}	0.120±0.005^{d}	0.166±0.012^{b}	0.175±0.009^{ab}
**Sugars**						
Fucose	0.49±0.01^{d}	0.56±0.02^{d}	1.61±0.03^{a}	1.33±0.06^{b}	0.93±0.03^{c}	1.60±0.05^{a}
1-O-methyl-alpha-D-mannopyranoside	0.32±0.02^{b}	0.40±0.02^{a}	0.32±0.00^{b}	0.29±0.01^{b}	0.25±0.01^{c}	0.30±0.01^{b}
1-O-methyl-alpha-D-glucopyranoside	0.54±0.02^{b}	0.48±0.02^{b}	0.69±0.00^{a}	0.67±0.03^{a}	0.67±0.03^{a}	0.48±0.04^{b}
Maltose	0.65±0.01^{bc}	0.67±0.03^{bc}	0.74±0.01^{a}	0.78±0.04^{a}	0.63±0.03^{c}	0.71±0.01^{ab}
alpha,alpha'-D-trehalose	0.07±0.00^{d}	0.10±0.01^{b}	0.08±0.00^{cd}	0.16±0.01^{a}	0.17±0.01^{a}	0.10±0.00^{bc}
Isomaltose	0.05±0.00^{d}	0.02±0.00^{c}	0.06±0.00^{d}	0.16±0.01^{a}	0.13±0.01^{b}	0.10±0.00^{c}
Raffinose	0.21±0.01^{c}	0.32±0.02^{c}	0.22±0.01^{c}	1.12±0.09^{b}	1.71±0.15^{a}	1.04±0.03^{b}
Xylose	2.46±0.18^{d}	2.13±0.08^{d}	3.66±0.13^{c}	11.15±0.73^{a}	8.48±0.15^{b}	10.61±0.31^{a}
Fructose	64.87±3.41^{d}	64.79±2.86^{d}	73.07±2.30^{cd}	98.23±3.54^{b}	119.48±4.56^{a}	79.98±1.99^{c}
Glucose	235.54±11.29^{c}	239.83±10.60^{c}	252.32±8.06^{c}	301.94±12.21^{b}	387.11±17.21^{a}	225.55±7.57^{c}
Sucrose	185.14±7.46^{b}	254.33±9.01^{a}	175.06±8.19^{bc}	165.31±6.53^{bc}	152.88±9.07^{c}	177.94±5.84^{b}
**Sugar-alcohols**						
Maltitol	0.18±0.01^{b}	0.17±0.01^{b}	0.21±0.00^{a}	0.22±0.01^{a}	0.17±0.01^{b}	0.21±0.00^{a}
Sorbitol	10.23±0.84^{a}	11.91±0.66^{a}	7.43±0.23^{b}	10.58±0.62^{a}	7.23±0.86^{b}	6.21±0.33^{b}
Myo-inositol	11.56±0.39^{a}	11.10±0.56^{a}	10.89±0.39^{a}	5.91±0.25^{b}	5.22±0.47^{bc}	4.34±0.13^{c}
**Organic acids**						
Pyruvic acid	0.101±0.003^{a}	0.090±0.004^{b}	0.088±0.003^{b}	0.039±0.002^{c}	0.028±0.001^{d}	0.032±0.001^{cd}
Glyceric acid	0.078±0.006^{b}	0.060±0.002^{de}	0.094±0.003^{a}	0.073±0.004^{bc}	0.052±0.001^{e}	0.064±0.003^{cd}
Succinic acid	0.35±0.01^{a}	0.20±0.01^{b}	0.32±0.01^{a}	0.18±0.01^{b}	0.19±0.03^{b}	0.17±0.01^{b}
Fumaric acid	0.050±0.005^{b}	0.058±0.000^{a}	0.043±0.001^{c}	0.025±0.001^{de}	0.024±0.001^{e}	0.031±0.001^{d}
Maleic acid	0.024±0.001^{ab}	0.026±0.001^{a}	0.027±0.001^{a}	0.026±0.001^{a}	0.022±0.001^{b}	0.025±0.001^{a}
Nicotinic acid	0.050±0.001^{a}	0.044±0.001^{c}	0.046±0.001^{bc}	0.048±0.001^{ab}	0.038±0.001^{d}	0.047±0.001^{bc}
2-oxo-glutaric acid	0.189±0.031^{a}	0.129±0.011^{b}	0.123±0.006^{b}	0.052±0.006^{c}	0.034±0.004^{c}	0.044±0.003^{c}
Citric acid	9.45±0.52^{b}	4.40±0.27^{d}	11.19±0.20^{a}	6.22±0.33^{c}	2.85±0.19^{e}	7.01±0.25^{c}
Galacturonic acid	0.121±0.014^{c}	0.152±0.009^{c}	0.199±0.016^{c}	0.312±0.029^{c}	2.169±0.362^{b}	2.658±0.051^{a}
2-amino-2-deoxy-D-gluconic acid	0.056±0.006^{a}	0.043±0.004^{ab}	0.053±0.007^{ab}	0.056±0.009^{a}	0.057±0.003^{a}	0.037±0.001^{b}
cis-3-caffeoyl-quinic acid	0.036±0.002^{c}	0.060±0.004^{a}	0.047±0.003^{b}	0.048±0.002^{b}	0.050±0.002^{b}	0.049±0.003^{b}
trans-3-caffeoyl-quinic acid	0.029±0.002^{c}	0.047±0.004^{a}	0.040±0.003^{ab}	0.039±0.002^{b}	0.041±0.001^{ab}	0.041±0.003^{ab}
Malic acid	81.55±4.29^{a}	85.87±1.26^{a}	85.53±2.26^{a}	55.06±3.1^{b}	42.83±1.83^{c}	49.72±1.47^{bc}
**Others**						
Phosphoric acid	15.42±0.39^{b}	15.42±0.15^{b}	17.67±0.24^{a}	13.69±0.48^{c}	14.93±0.48^{b}	13.60±0.57^{c}
Urea	0.049±0.003^{a}	0.052±0.004^{a}	0.034±0.002^{b}	0.034±0.001^{bc}	0.026±0.002^{c}	0.034±0.003^{b}
Putrescine	0.65±0.09^{a}	0.66±0.01^{a}	0.11±0.00^{b}	0.20±0.00^{b}	0.10±0.01^{b}	0.18±0.01^{b}
